# Significance of chemotherapy-free interval and tumor regression grade in patients with recurrent esophageal squamous cell carcinoma receiving chemotherapy with fluorouracil and platinum after esophagectomy following preoperative chemotherapy

**DOI:** 10.1007/s10388-021-00885-3

**Published:** 2021-10-05

**Authors:** Mashiro Okunaka, Daisuke Kotani, Ken Demachi, Hisashi Fujiwara, Shingo Sakashita, Takayuki Yoshino, Takeo Fujita, Takashi Kojima

**Affiliations:** 1 Department of Pharmacy, National Cancer Center Hospital East, Kashiwa, Japan; 2 Department of Gastrointestinal Oncology, National Cancer Center Hospital East, 6-5-1 Kashiwanoha, Kashiwa, Chiba 277-8577 Japan; 3 Division of Esophageal Surgery, National Cancer Center Hospital East, Kashiwa, Japan; 4 Division of Pathology, Exploratory Oncology Research and Clinical Trial Center, National Cancer Center Hospital East, Kashiwa, Japan

**Keywords:** Esophageal squamous cell carcinoma, Chemotherapy, Chemotherapy-free interval, Tumor regression grade

## Abstract

**Background:**

In Japan, standard treatment for locally advanced esophageal squamous cell carcinoma (ESCC) includes preoperative chemotherapy with fluorouracil plus cisplatin followed by esophagectomy. However, its efficacy is unclear in patients with recurrent disease with < 6 months of chemotherapy-free interval (CFI) after preoperative chemotherapy followed by esophagectomy and in those with ≥ 6 months of CFI and poor pathological response to prior preoperative chemotherapy.

**Method:**

We retrospectively evaluated the efficacy of fluorouracil plus platinum in patients with recurrent ESCC who received preoperative chemotherapy followed by curative esophagectomy.

**Results:**

Among 105 patients with recurrent ESCC after preoperative chemotherapy followed by esophagectomy, a total of 55 patients received fluorouracil plus platinum for recurrent disease. Patients with a CFI < 6 months (*n* = 20) had significantly shorter overall survival (OS) (median, 7.1 vs 14.5 months, *P* = 0.008) compared with those with a CFI ≥ 6 months (*n* = 35). Multivariate analysis showed that OS was worse in patients with a CFI < 6 months or a tumor regression grade (TRG) ≤ 1a. Furthermore, in patients with a CFI ≥ 6 months, TRG ≤ 1a was associated with significantly shorter OS (11.1 months vs. not reached, *P* = 0.001).

**Conclusion:**

Fluorouracil plus platinum was ineffective for recurrent ESCC in patients with a CFI < 6 months and in those with a CFI ≥ 6 months and a TRG ≤ 1a. Alternate regimens including nivolumab or pembrolizumab might be considered for the treatment for recurrence in these patients.

**Supplementary Information:**

The online version contains supplementary material available at 10.1007/s10388-021-00885-3.

## Introduction

Esophageal cancer remains the sixth leading cause of cancer mortality worldwide [1, 2]. Squamous cell carcinoma (SCC) accounts for over 90% of all esophageal cancer cases in East Asia, whereas adenocarcinoma is the dominant histological subtype in Western countries [3]. The Japanese Clinical Oncology Group (JCOG) 9907 trial, which compared preoperative chemotherapy with fluorouracil plus cisplatin followed by esophagectomy and esophagectomy followed by postoperative chemotherapy in patients with locally advanced esophageal SCC (ESCC), demonstrated the survival benefit of preoperative chemotherapy (5-year overall survival [OS] 55% vs 43%, hazard ratio [HR] 0.73, *P* = 0.04) [4]. Based on the JCOG9907 trial, preoperative chemotherapy with fluorouracil plus cisplatin is a standard treatment regimen for locally advanced ESCC in Japan. Meanwhile, in the phase III (CROSS trial) conducted in Western countries, preoperative chemoradiotherapy consisting of carboplatin plus paclitaxel and preoperative concurrent radiotherapy with 41.4 Gy improved OS compared to surgery alone for locally advanced esophageal or esophagogastric cancer including both SCC and adenocarcinoma histologies [5, 6]. Therefore, preoperative chemotherapy or chemoradiotherapy followed by esophagectomy is a standard treatment for locally advanced ESCC. However, approximately 40–50% of patients recur within 3 years after esophagectomy with preoperative chemotherapy or chemoradiotherapy [4, 6].

Fluoropyrimidine plus oxaliplatin/cisplatin is the preferred first-line chemotherapy regimen for recurrent or metastatic esophageal or esophagogastric cancer including SCC and adenocarcinoma [2]. In gastric cancer patients with recurrent disease after curative surgery within 6 months after adjuvant S-1 chemotherapy, the response rate to treatment with S-1 plus cisplatin is only 5%, suggesting that S-1 plus cisplatin should be considered for patients with a recurrence-free interval ≥ 6 months [7]. Meanwhile, the only retrospective small cohort study on recurrent ESCC suggested that seven patients with a chemotherapy-free interval (CFI) < 6 months had a lack of sensitivity to the fluorouracil plus cisplatin regimen, which was administered as prior preoperative chemotherapy [8]. Since almost all clinical trials on first-line chemotherapy for recurrent or metastatic esophageal or esophagogastric cancer including SCC exclude patients with recurrent disease and a CFI < 6 months [9–11], the efficacy of fluorouracil plus platinum for recurrent disease in this patient population is unclear.

Tumor regression grade (TRG) has been reported as a prognostic marker for disease-free survival and OS in patients with ESCC as well as adenocarcinoma [12]. However, the association of TRG with the efficacy of fluorouracil plus platinum on recurrent disease and in patients having a CFI > 6 months with unfavorable TRG is unclear.

Therefore, the present study aimed to investigate the efficacy of fluorouracil plus platinum according to CFI and TRG in patients with recurrent ESCC who received preoperative chemotherapy followed by curative esophagectomy.

## Methods

### Study design and patients

This retrospective study was designed to evaluate the efficacy of fluorouracil plus platinum regimen for patients with recurrent ESCC who received identical preoperative chemotherapy followed by curative esophagectomy. The study protocol was approved by the institutional review board of the National Cancer Center Hospital East (2020-589). Informed consent requirement was waived due to the retrospective observational design of the study, with opt-out opportunity provided at the institution’s website.

The eligibility criteria were as follows: age, ≥ 20 years; diagnosis of histologically proven recurrent ESCC after preoperative chemotherapy with fluorouracil plus cisplatin and subsequent curative esophagectomy (R0 or R1 according to the AJCC Cancer Staging Manual) [13]; Eastern Cooperative Oncology Group (ECOG) performance status (PS) score, 0–2; adequate bone marrow and organ function to receive chemotherapy; evaluable lesions according to the Response Evaluation Criteria in Solid Tumors version 1.1 [14]; and treatment with fluorouracil plus platinum for recurrent ESCC between June 1, 2008 and March 31, 2020 at the National Cancer Center Hospital East.

### Study procedures

Preoperative chemotherapy comprised two cycles of fluorouracil (800 mg/m^2^, days 1–5) plus cisplatin (80 mg/m^2^, day 1) every 3 weeks or three cycles of fluorouracil (750 mg/m^2^, days 1–5), cisplatin (70 mg/m^2^, day 1), and docetaxel (70 mg/m^2^, day 1) every 3 weeks. Surgery was performed by total or subtotal thoracic esophagectomy with three-field lymphadenectomy. Chemotherapy for recurrent disease comprised fluorouracil (800 mg/m^2^, days 1–5) plus cisplatin (80 mg/m^2^, day 1) every 4 weeks, fluorouracil (800 mg/m^2^, days 1–5) plus nedaplatin (80–90 mg/m^2^, day 1) every 4 weeks, and oxaliplatin (85 mg/m^2^, day 1), leucovorin (200 mg/m^2^, day 1), and fluorouracil (400 mg/m^2^ intravenous bolus on day 1 and 2400 mg/m^2^ by continuous 46-hinfusion on day 1) every 2 weeks. Dose modification and treatment interruption were determined by each investigator.

The following baseline characteristics were collected for each patient: age, sex, ECOG PS score, clinical stage at initial diagnosis, preoperative chemotherapy, CFI after last administration of preoperative chemotherapy, TRG, and recurrence sites.

### Outcomes

The initiation of study treatment was defined as the start date of palliative chemotherapy for recurrent ESCC. Efficacy endpoints included progression-free survival (PFS), defined as the time interval from the initiation of study treatment to disease progression or death due to any cause; OS, defined as the time interval from the initiation of study treatment to death due to any cause; overall response rate (ORR), defined as the proportion of patients with complete or partial response to the study treatment; disease control rate (DCR), defined as the proportion of patients with complete or partial response plus stable disease lasting for > 6 weeks from the initiation of study treatment; and CFI, defined as the time interval from last administration of preoperative chemotherapy to recurrence. Tumor response was assessed by each physician using the Response Evaluation Criteria in Solid Tumors version 1.1 every 8 weeks from the initiation of treatment until disease progression. Clinical and pathological stage was defined according to UICC-TNM 7th edition. Tumor regression was graded according to the Japanese Classification of Esophageal Cancer as follows: grade 0, no recognizable cytological or histological therapeutic effect; grade 1a, viable cancer cells accounting for two-thirds of tumor; grade 1b, viable cancer cells accounting for one-third or more but less than two-thirds of tumor; grade 2, viable cancer cells accounting for less than one-third of tumor; and grade 3, no evidence of viable cancer cells [15].

### Statistical analysis

PFS and OS were determined using Kaplan–Meier estimates, and rates between the treatment groups were compared using the log-rank test with a two-sided *P* value of 0.05. HRs and corresponding 95% confidence intervals (CIs) were determined using the Cox proportional hazards model. Comparisons of ORR, DCR, and safety outcomes between the treatment groups were performed using Fisher’s exact test. Follow-up time was defined as the time from the initiation of study treatment until last follow-up for censored cases. The Cox regression model was used to assess the impact of CFI and TRG on PFS and OS, with adjustment for other factors which were considered to be associated with outcomes based on univariate log-rank test. Statistical analyses were performed using SPSS version 22.0 (IBM, Armonk, NY, USA), and a two-sided *P* value of < 0.05 denoted statistical significance.

## Results

### Patients

**Fig. 1:**
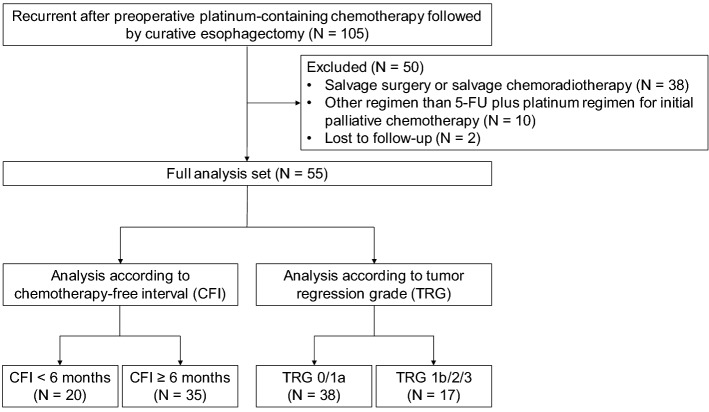
Flow diagram

During the study period, there were 105 patients with recurrent ESCC after preoperative chemotherapy followed by curative esophagectomy. Fifty patients were excluded due to salvage surgery or chemoradiotherapy (*N* = 38), other regimen than 5-FU plus platinum for initial palliative chemotherapy (*N* = 10), and lost to follow-up (*N* = 2). Finally, 55 patients who received fluorouracil plus platinum for recurrent disease were included in the full analysis set (Fig. 1). Among them, 26 (47.3%) and 29 (52.7%) patients received fluorouracil plus cisplatin and combination regimen with fluorouracil, cisplatin, and docetaxel, respectively, as preoperative chemotherapy; 39 of the 55 patients (70.9%) completed the planned preoperative chemotherapy. Sixteen patients (29.1%) discontinued preoperative chemotherapy due to disease progression (*n* = 10) or chemotherapeutic toxicity (*n* = 6). Twenty (36.4%) and 35 patients (63.6%) had a CFI of < 6 and ≥ 6 months, respectively. Meanwhile, TRG 0/1a and TRG 1b/2/3 were in 38 (69.1%) and 17 patients (30.9%), respectively. Chemotherapy for recurrent disease included fluorouracil plus cisplatin, fluorouracil plus nedaplatin, and combination of oxaliplatin, leucovorin, and fluorouracil in 37 (67.3%), 14 (25.5%), and 4 (7.3%), respectively.

Table 1 presents the comparison of baseline characteristics at the start of systemic chemotherapy for recurrent ESCC in patients with a CFI of < 6 and ≥ 6 months after the last administration of preoperative chemotherapy. There were no significant differences in baseline characteristics, including age, sex, ECOG PS score, clinical stage, pathological stage, TRG, and number and location of sites of recurrence, at the time of recurrent ESCC diagnosis. Regarding preoperative chemotherapy, the rate of combination chemotherapy with fluorouracil, cisplatin, and docetaxel was higher in patients with a CFI < 6 months than in those with a CFI ≥ 6 months (70.0% vs 42.9%, *P* = 0.048). Likewise, Table 2 presents the baseline characteristics according to a TRG 0/1a and TRG 1b/2/3 for preoperative chemotherapy. There were also no significant differences in baseline characteristics other than preoperative chemotherapy regimen (combination chemotherapy with fluorouracil, cisplatin, and docetaxel, 39.5% vs 82.4%, *P* = 0.004).

**Table 1:** Patient characteristics according to CFI

	CFI < 6 months		CFI ≥ 6 months		*P* value
	*N* = 20	%	*N* = 35	%	
Age					
Median (range)	64 (44 − 79)		68 (50–78)		0.110
≥ 65 years old	9	45.0	20	57.1	0.415
Gender					
Male	16	80.0	30	85.7	0.709
ECOG PS					
0	16	80.0	26	74.3	0.749
> 1	4	20.0	9	25.7	
cStage					
I	0	0.0	3	8.6	0.326
II	3	15.0	10	28.6	
III	11	55.0	16	45.7	
IV	6	30.0	6	17.1	
Preoperative chemotherapy					
FP	6	30.0	20	57.1	0.048
DCF	14	70.0	15	42.9	
Extent of resection					
R0	16	80.0	35	100.0	0.014
R1	4	20.0	0	0.0	
ypStage					
I	0	0.0	6	17.1	0.121
II	5	25.0	10	28.6	
III	9	45.0	15	42.9	
IV	6	30.0	4	11.4	
TRG					
0/1a	15	75.0	23	65.7	0.555
1b/2/3	5	25.0	12	34.3	
Number of recurrent site					
1	10	50.0	13	37.1	0.403
> 2	10	50.0	22	62.9	
Site of recurrence					
Liver	7	35.0	9	25.7	0.543
Lung	6	30.0	14	40.0	0.565
Lymph node	12	60.0	25	71.4	0.551
Bone	4	20.0	8	22.9	1.000
Others	6	30.0	9	25.7	0.761

*ECOG* *PS* Eastern Cooperative Oncology Group performance status

**Table 2:** Patient characteristics according to TRG

	TRG 0/1a		TRG 1b/2/3		*P* value
	*N* = 38	%	*N* = 17	%	
Age					
Median (range)	65 (44 − 79)		64 (44–78)		0.539
≥ 65 years old	20	52.6	9	52.9	1.000
Gender					
Male	33	86.8	13	76.5	0.435
ECOG PS					
0	29	76.3	13	76.5	1.000
≥ 1	9	23.7	4	23.5	
cStage					
I	3	7.9	0	0.0	0.066
II	12	31.6	1	5.9	
III	17	44.7	10	58.8	
IV	6	15.8	6	35.3	
Preoperative chemotherapy					
FP	23	60.5	3	17.6	0.004
DCF	15	39.5	14	82.4	
Extent of resection					
R0	34	89.5	17	100.0	0.299
R1	4	10.5	0	0.0	
ypStage					
I	3	7.9	3	17.6	0.053
II	8	21.1	7	41.2	
III	21	55.3	3	17.6	
IV	6	15.8	4	23.5	
CFI					
< 6 months	15	39.5	5	29.4	0.555
≥ 6 months	23	60.5	12	70.6	
Number of recurrent site					0.376
1	14	36.8	9	52.9	
≥ 2	24	63.2	8	47.1	
Site of recurrence					
Liver	10	26.3	6	35.3	0.533
Lung	13	34.2	7	41.2	0.763
Lymph node	28	73.7	9	52.9	0.213
Bone	9	23.7	3	17.6	0.735
Others	11	28.9	4	23.5	0.754

*ECOG* *PS* Eastern Cooperative Oncology Group performance status

### Efficacy

Fifty-four patients discontinued the regimen with fluorouracil plus platinum for recurrent disease due to disease progression (*n* = 50) or chemotherapeutic toxicity (*n* = 4), and the study treatment was ongoing in one patient at last follow-up. The median PFS was 1.9 (95% CI 1.6–2.2) months (Supplemental Fig. 1a). Eleven patients achieved complete response (*n* = 1) or partial response (*n* = 10), and 12 patients were determined to have stable disease, with an ORR of 20.0% and DCR of 41.8%. During the study period, 38 patients (69.1%) died. The median OS was 11.4 (95% CI 7.5–15.3) months during a median follow-up period of 21.7 (95% CI 19.2–24.2) months (Supplemental Fig. 1b). Thirty-five patients (63.6%) received subsequent antitumor therapy, including taxanes (*n* = 31), investigational agents in clinical trials (*n* = 8), and anti-programmed cell death 1 antibody (*n* = 6).

### Impact of CFI and TRG

**Fig. 2:**
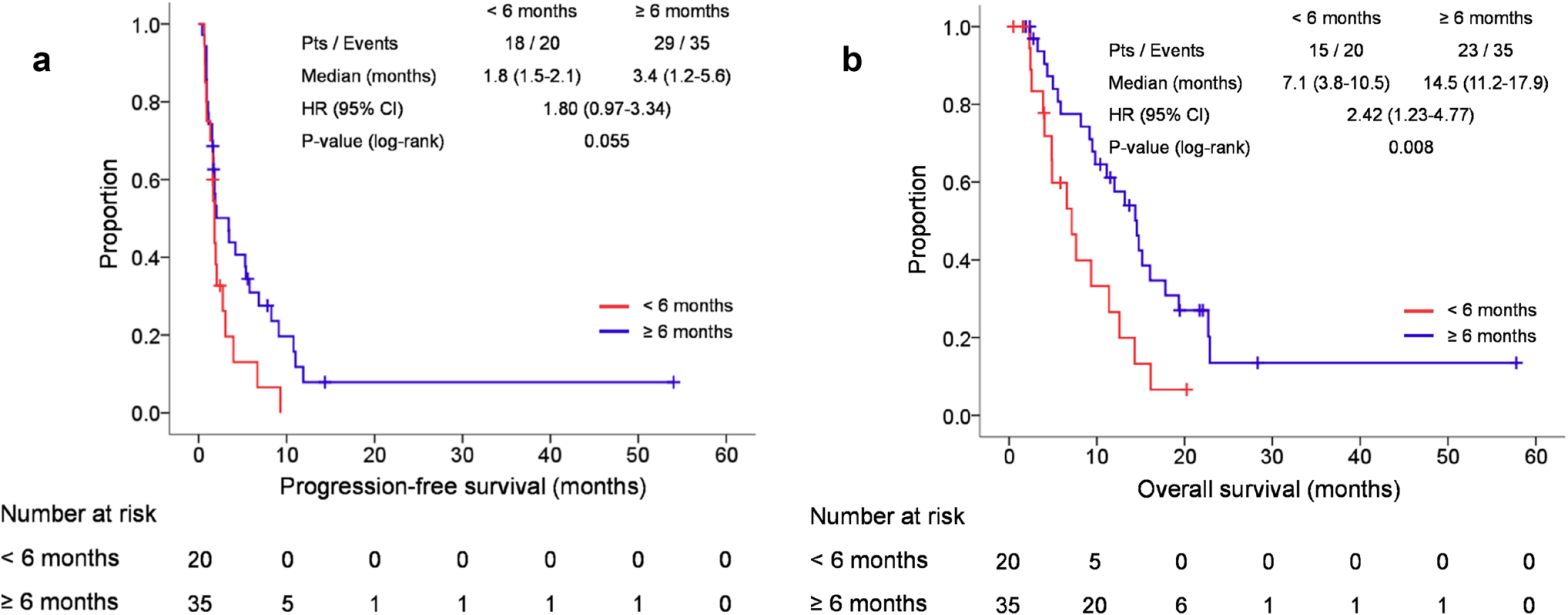
**a** Kaplan–Meier estimates of progression-free survival according to CFI. CFI, chemotherapy-free interval; HR, hazard ratio. **b** Kaplan–Meier estimates of overall survival according to CFI. *CFI* chemotherapy-free interval; *HR* hazard ratio

Patients with a CFI < 6 months had shorter PFS with marginal significance (median 1.8 vs. 3,4 months, HR 1.80, 95% CI 0.97–3.34, *P* = 0.055) and significantly shorter OS (median 7.1 vs. 14.5 months, HR 2.42, 95% CI 1.23–4.77, *P* = 0.008) compared with those with a CFI ≥ 6 months (Fig. 2). In addition, albeit not statistically significant, the ORR was lower in patients with a CFI < 6 months than in those with a CFI ≥ 6 months (10.0% vs. 25.7%, *P* = 0.293) (Table 3).

**Table 3:** Response rate according to CFI and TRG

	CFI < 6 months				*P* value	CFI ≥ 6 months				*P* value
	Grade 0/1a		Grade 1b/2/3			Grade 0/1a		Grade 1b/2/3		
	*N* = 15	%	*N* = 5	%		*N* = 23	%	*N* = 12	%	
CR	0	0.0	0	0.0		0	0.0	1	8.3	
PR	1	6.7	1	20.0		2	8.7	6	50.0	
SD	3	20.0	1	20.0		5	21.7	3	25.0	
PD	11	73.3	3	60.0		16	69.6	2	16.7	
ORR	1	6.7	1	20.0	0.447	2	8.7	7	58.3	0.003
DCR	4	26.7	2	40.0	0.613	7	30.4	10	83.3	0.005

*CR* complete response, *PR* partial response, *SD* stable disease, *PD* progressive disease, *ORR* objective response rate, *DCR* disease control rate

**Fig. 3:**
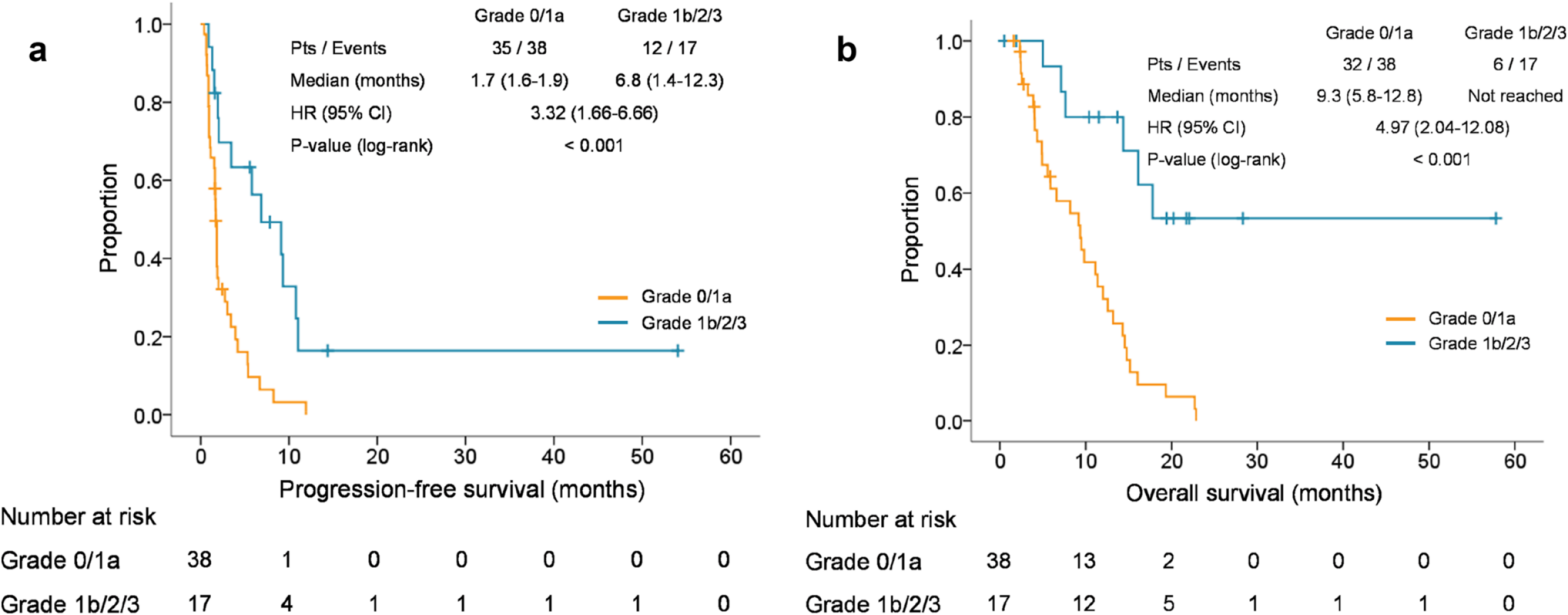
**a** Kaplan–Meier estimates of progression-free survival according to TRG. *TRG* tumor regression grade; *HR* hazard ratio. **b** Kaplan–Meier estimates of overall survival according to TRG. *TRG* tumor regression grade; *HR* hazard ratio

TRG ≤ 1a was associated with significantly shorter PFS (median 1.7 vs. 6.8 months, HR 3.32, 95% CI 1.66–6.66, *P* < 0.001), shorter OS (median 9.3 months vs. not reached, HR 4.97, 95% CI 2.04–12.08, *P* < 0.001) and lower ORR (7.9% vs. 47.1%, *P* < 0.001) compared with TRG ≥ 1b (Fig. 3).

By multivariate analysis for PFS, TRG ≤ 1a was independently associated with poor outcome (Table 4). Additionally, in multivariate analyses for OS, only TRG ≤ 1a and CFI < 6 months were independent poor prognostic factors (Table 5). Furthermore, even after excluding ten patients who were experienced disease progression during preoperative chemotherapy, TRG ≤ 1a and CFI < 6 months were still independent poor prognostic factors for OS in multivariate analysis (Supplemental Table 1).

**Table 4:** Univariate and multivariate analysis for progression-free survival

Variables	Category	Univariate analysis		Multivariate analysis	
		HR (95% CI)	*P* value	HR (95% CI)	*P* value
Age	≥ 65 vs < 65	0.743 (0.414–1.335)	0.320		
Gender	Male vs female	0.681 (0.316–1.468)	0.326		
ECOG PS	0 vs ≥ 1	0.952 (0.493–1.838)	0.882		
cStage	I-II vs III-IV	1.069 (0.576–1.983)	0.833		
Preoperative chemotherapy	FP vs DCF	1.155 (0.650–2.049)	0.624		
Extent of resection	R0 vs R1	0.769 (0.235–2.514)	0.664		
ypStage	I-II vs III-IV	0.663 (0.364–1.208)	0.179	0.965 (0.501–1.862)	0.916
TRG	0/1a vs 1b/2/3	3.320 (1.656–6.656)	0.001	3.965 (1.843–8.529)	< 0.001
CFI (months)	< 6 vs ≥ 6	1.801 (0.972–3.339)	0.062	1.825 (0.956–3.483)	0.068
Number of recurrent sites	≥ 2 vs 1	2.326 (1.256–4.310)	0.007	1.946 (0.789–4.785)	0.149
Site of recurrence	Liver	0.975 (0.520–1.828)	0.937		
	Lung	1.576 (0.866–2.868)	0.136	1.812 (0.934–3.516)	0.079
	Lymph node	1.948 (1.022–3.714)	0.043	1.228 (0.485–3.108)	0.665
	Bone	1.032 (0.524–2.029)	0.928		
	Others	1.420 (0.723–2.789)	0.309

**Table 5:** Univariate and multivariate analysis for overall survival

Variables	Category	Univariate analysis		Multivariate analysis	
		HR (95% CI)	*P* value	HR (95% CI)	*P* value
Age	≥ 65 vs < 65	1.137 (0.597–2.166)	0.696		
Gender	Male vs Female	1.253 (0.486–3.236)	0.640		
ECOG PS	0 vs ≥ 1	0.932 (0.447–1.939)	0.850		
cStage	I-II vs III-IV	1.206 (0.615–2.364)	0.586		
Preoperative chemotherapy	FP vs DCF	1.326 (0.696–2.532)	0.390		
Extent of resection	R0 vs R1	0.270 (0.092–0.790)	0.017	0.787 (0.237–2.614)	0.696
ypStage	I-II vs III-IV	0.609 (0.313–1.186)	0.145	0.941 (0.465–1.906)	0.866
TRG	0/1a vs 1b/2/3	4.968 (2.044–12.075)	< 0.001	5.235 (2.079–13.184)	< 0.001
CFI (months)	< 6 vs ≥ 6	2.421 (1.229–4.766)	0.011	2.590 (1.193–5.619)	0.016
Number of recurrent sites	≥ 2 vs 1	1.279 (0.666–2.451)	0.461		
Site of recurrence	Liver	0.841 (0.408–1.733)	0.639		
	Lung	0.894 (0.457–1.750)	0.743		
	Lymph node	1.454 (0.710–2.976)	0.306		
	Bone	1.026 (0.449–2.346)	0.951		
	Others	1.501 (0.721–3.125)	0.277		

**Fig. 4:**
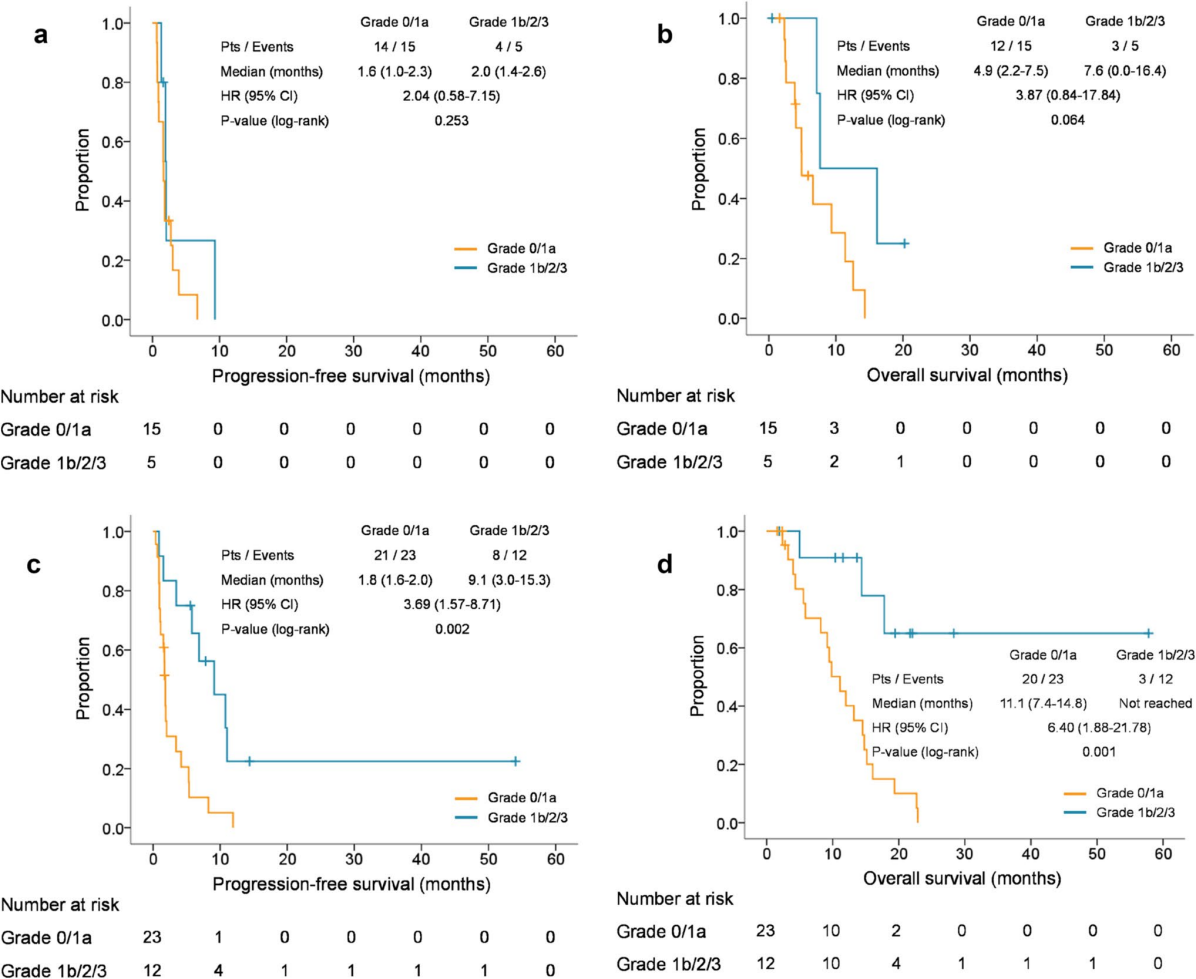
**a** Kaplan–Meier estimates of progression-free survival according to TRG in patients with a CFI < 6 months. *CFI* chemotherapy-free interval; *TRG* tumor regression grade; HR, hazard ratio. **b** Kaplan–Meier estimates of overall survival according to TRG in patients with a CFI < 6 months. *CFI* chemotherapy-free interval; *TRG* tumor regression grade; *HR* hazard ratio. **c** Kaplan–Meier estimates of progression-free survival according to TRG in patients with a CFI ≥ 6 months. *CFI* chemotherapy-free interval; *TRG* tumor regression grade; *HR*, hazard ratio. **d** Kaplan–Meier estimates of overall survival according to TRG in patients with a CFI ≥ 6 months *CFI* chemotherapy-free interval; *TRG* tumor regression grade; *HR* hazard ratio

Among the patients with a CFI < 6 months, there were no significant differences in PFS and OS between the patients with a TRG ≤ 1a and ≥ 1b (PFS, median 1.6 vs. 2.0 months, respectively, HR 2.04, 95% CI 0.58–7.15, *P* = 0.253; OS, median 4.9 vs. 7.6 months, respectively, HR 3.87, 95% CI 0.84–17.84, *P* = 0.064) (Fig. 4). Furthermore, although a trend for lower response was observed in patients with a TRG ≤ 1a compared to in those with a TRG ≥ 1b (6.7% vs 20.0%), there was no significant difference (*P* = 0.447) (Table 3). Conversely, among the patients with a CFI ≥ 6 months, the patients with a TRG ≤ 1a had significantly shorter PFS and OS compared to those with a TRG ≥ 1b (PFS, median 1.8 vs. 9.1 months, HR 3.69, 95% CI 1.57–8.71, *P* = 0.002; OS, median 11.1 months vs. not reached, HR 6.40, 95% CI 1.88–21.78, *P* = 0.001) (Fig. 4). Furthermore, the ORR was significantly lower in patients with a TRG ≤ 1a compared to those with a TRG ≥ 1b (8.7% vs. 58.3%, *P* = 0.003) (Table 3).

## Discussion

The results of this retrospective study, which evaluated the efficacy of combination chemotherapy with fluorouracil plus platinum in patients with recurrent ESCC who previously received preoperative chemotherapy followed by curative esophagectomy, indicate that CFI and TRG should be taken into consideration in deciding on whether patients should receive chemotherapy with fluorouracil plus platinum for recurrent ESCC after esophagectomy with preoperative chemotherapy.

In patients with ESCC, the value of palliative chemotherapy remains unclear. The European Society of Medical Oncology clinical practice guidelines indicate that best supportive care or palliative monotherapy should be considered in these patients [16]. More recently, although the pan-Asian adapted European Society of Medical Oncology clinical practice guidelines referred that combination chemotherapy is the preferred option for fit patients [17], standard chemotherapy regimen was not defined in the guideline. Conversely, in the recent KEYNOTE-590 trial, which demonstrated the superiority of pembrolizumab plus chemotherapy over chemotherapy alone as first-line chemotherapy for esophageal cancer, combination chemotherapy with fluorouracil and cisplatin was considered as a standard first-line chemotherapy for advanced ESCC [18]. Therefore, platinum-based combination chemotherapy has been a community standard as first-line chemotherapy for advanced ESCC despite insufficient evidence. Furthermore, most clinical trials, including the KEYNOTE-590 trial, excluded patients with a CFI < 6 months. However, evidence is lacking regarding the exclusion of patients with recurrent disease and a CFI < 6 months from receiving first-line chemotherapy [9–11].

The present study demonstrated that the outcomes were unfavorable in patients with a CFI < 6 months compared to those with a CFI ≥ 6 months. Considering the significantly shorter OS and relatively lower ORR of 10.5% and a shorter median PFS of 1.8 months in patients with CFI < 6 months, other regimens should be considered as alternative chemotherapy in those patients. In fact, the ATTRACTION-3 [19] and KEYNOTE-181 [20] trials, which evaluated nivolumab and pembrolizumab, respectively, in a second-line setting for esophageal cancer, included patients with disease recurrence within 6 months from the last administration of preoperative systemic chemotherapy or chemoradiotherapy. These trials revealed that the ORRs were 19% and 22% with nivolumab in all ESCC patients and pembrolizumab in patients with ESCC who had a PD-L1 combined positive score ≥ 10, respectively.

We also demonstrated the significance of TRG in patients receiving chemotherapy for recurrent ESCC. TRG ≤ 1a was associated with significantly shorter PFS and OS and was an independent poor prognostic factor for PFS and OS by univariate and multivariate analyses. Our study suggested that patients with TRG ≤ 1a were primary insensitive to fluorouracil plus platinum regimens. In fact, only two of the patients with a CFI ≥ 6 months and TRG ≤ 1a partial responded to the study treatment (ORR of 8.7%). Considering an ORR of 29.3% for first-line chemotherapy in the control group of the KEYNOTE-590 trial [18], alternative regimens including nivolumab or pembrolizumab might be considered in these patients. In contrast, for patients with a CFI < 6 months and TRG ≥ 1b, although only one of the five patients responded to the study treatment (ORR of 20.0%), fluorouracil plus platinum is not generally recommended based on the extremely short median PFS of 2.0 months.

The present study has several limitations. First, this was a non-randomized retrospective study with a limited sample size performed in a single institution. In particular, preoperative chemotherapy regimens were imbalanced between the groups. However, we performed the multivariate analyses, confirming the independent prognostic factor of CFI and TRG for OS in patients receiving palliative chemotherapy for recurrent ESCC. Second, treatment after recurrence was selected individually by each physician. Patients who received other regimens for recurrent disease might not be fit for combination regimen or may experience a clinically poor response to preoperative treatment with a regimen containing fluorouracil and platinum. Finally, all patients received preoperative chemotherapy, not chemoradiotherapy, as a standard preoperative treatment for ESCC in Japan.

In conclusion, in this largest cohort study to evaluate the efficacy of combination chemotherapy with fluorouracil plus platinum in patients with recurrent ESCC who previously received preoperative chemotherapy followed by curative esophagectomy, fluorouracil plus platinum for recurrent ESCC was ineffective in patients with a CFI < 6 months and in those with a CFI ≥ 6 months and a TRG ≤ 1a. These patients may be considered to receive alternative regimens including nivolumab and pembrolizumab for the treatment of recurrent disease.

## Supplementary Information

Below is the link to the electronic supplementary material.

Supplementary file1 (DOCX 64 KB)
