# Supplementary material for: Significance of chemotherapy-free interval and tumor regression grade in patients with recurrent esophageal squamous cell carcinoma receiving chemotherapy with fluorouracil and platinum after esophagectomy following preoperative chemotherapy
Source: Esophagus. 2021 Oct 5;19(2):240–9. doi: 10.1007/s10388-021-00885-3 (PMC8921032; doi:10.1007/s10388-021-00885-3)
Supplement: Supplementary file 1 — Supplementary file1 (DOCX 64 KB) [file 10388_2021_885_MOESM1_ESM.docx]

Supplemental Figure 1a　 Kaplan-meier estimates of progression-free survival in all patients

Supplemental Figure 1b　 Kaplan-meier estimates of overall survival in all patients

Supplemental Table 1　 Univariate and Multivariate analysis for overall survival excluding patients who refractory to preoperative chemotherapy

| Variables | category | Univariate analysis | | Multivariate analysis | |
| --- | --- | --- | --- | --- | --- |
|  |  | HR (95% CI) | P value | HR (95% CI) | P value |
| Age | ≥ 65 vs < 65 | 1.126 (0.537-2.361) | 0.753 |  |  |
| Gender | Male vs Female | 1.650 (0.496-5.495) | 0.414 |  |  |
| ECOG PS | 0 vs ≥ 1 | 0.902 (0.380-2.140) | 0.741 |  |  |
| cStage | 0-II vs III-IV | 1.280 (0.593-2.762) | 0.530 |  |  |
| Preoperative chemotherapy | FP vs DCF | 1.209 (0.579-2.525) | 0.613 |  |  |
| Extent of resection | R0 vs R1 | 0.209 (0.068-0.645) | 0.006 | 0.736 (0.226-2.395) | 0.610 |
| ypStage | I-II vs III-IV | 0.620 (0.290-1.323) | 0.216 |  |  |
| TRG | 0/1a vs 1b/2/3 | 4.444 (1.787-11.050) | 0.001 | 5.427 (2.132-13.813) | < 0.001 |
| CFI (months) | < 6 vs ≥ 6 | 2.743 (1.260-5.971) | 0.011 | 2.799 (1.267-6.181) | 0.011 |
| Number of recurrent site | ≥ 2 vs 1 | 1.361 (0.648-2.857) | 0.416 |  |  |
| Site of recurrence | Liver | 0.886 (0.392-2.002) | 0.770 |  |  |
|  | Lung | 0.700 (0.297-1.647) | 0.413 |  |  |
|  | Lymph node | 1.211 (0.548-2.677) | 0.637 |  |  |
|  | Bone | 1.243 (0.523-2.954) | 0.622 |  |  |
|  | Others | 1.898 (0.847-4.254) | 0.120 | 1.054 (0.701-3.229) | 0.295 |
